# Distinct Type I Interferon Subtypes Differentially Stimulate T Cell Responses in HIV-1-Infected Individuals

**DOI:** 10.3389/fimmu.2022.936918

**Published:** 2022-07-13

**Authors:** Zehra Karakoese, Mara Schwerdtfeger, Christina B. Karsten, Stefan Esser, Ulf Dittmer, Kathrin Sutter

**Affiliations:** ^1^ Institute for Virology, University Medicine Essen, University of Duisburg-Essen, Essen, Germany; ^2^ Institute for Translational HIV Research, University Medicine Essen, University of Duisburg-Essen, Essen, Germany; ^3^ Department of Dermatology and Venerology, University Medicine Essen, Essen, Germany

**Keywords:** type I IFNs, HIV, LPMCs, CD8^+^ T cells, CD4^+^ T cells, NK cells

## Abstract

The expression of type I interferons (IFNs) is one of the immediate host responses during most viral infections. The type I IFN family consists of numerous highly conserved IFNα subtypes, IFNβ, and some others. Although these IFNα subtypes were initially believed to act interchangeably, their discrete biological properties are nowadays widely accepted. Subtype-specific antiviral, immunomodulatory, and anti-proliferative activities were reported explained by differences in receptor affinity, downstream signaling events, and individual IFN-stimulated gene expression patterns. Type I IFNs and increased IFN signatures potentially linked to hyperimmune activation of T cells are critically discussed for chronic HIV (human immunodeficiency virus) infection. Here, we aimed to analyze the broad immunological effects of specific type I IFN subtypes (IFNα2, IFNα14, and IFNβ) on T and NK cell subsets during HIV-1 infection *in vitro* and *ex vivo*. Stimulation with IFNα14 and IFNβ significantly increased frequencies of degranulating (CD107a^+^) gut-derived CD4^+^ T cells and blood-derived T and NK cells. However, frequencies of IFNγ-expressing T cells were strongly reduced after stimulation with IFNα14 and IFNβ. Phosphorylation of downstream molecules was not only IFN subtype-specific; also, significant differences in STAT5 phosphorylation were observed in both healthy peripheral blood mononuclear cells (PBMCs) and PBMCs of HIV-infected individuals, but this effect was less pronounced in healthy gut-derived lamina propria mononuclear cells (LPMCs), assuming cell and tissue specific discrepancies. In conclusion, we observed distinct type I IFN subtype-specific potencies in stimulating T and NK cell responses during HIV-1-infection.

## Introduction

The expression of type I interferons (IFNs) is one of the immediate host responses during most viral infections. The type I IFN family comprises multiple IFNα subtypes, which are highly conserved in their amino acid sequence. Despite their sequence homology, they all differ in their biological activity such as their antiviral, immunomodulatory, and anti-proliferative properties (1–5) possibly due to their different affinities to the type I IFN receptor (IFNAR) (6), as well as differences concerning downstream signaling events (1) leading to the induction of distinct IFN-stimulated gene (ISG) expression patterns (3, 4, 7).

Apart from its direct antiviral effect, IFNα also exerts diverse immunomodulatory properties with high therapeutic potential. Indeed, pegylated IFNα2 is used as clinical treatment in chronic Hepatitis B virus (HBV) patients with limited success resulting in up to 36% seroconversion of the HBV viral protein HBe and a loss of 11% of the HBV surface antigen HBsAg (8, 9). Unfortunately, IFN therapy is currently associated with disadvantages including the high variability of responses between patients and unfavorable side effects. However, the complete potential of IFNα for therapeutics is not fully understood yet. In-depths functional profiling of IFNα species, in particularly with regard to their ability to stimulate immune cells to eradicate viral reservoirs, might unravel new approaches to functional cures urgently needed to help individuals with chronic viral infections like HBV or Human immunodeficiency virus (HIV).

We could recently show in HBV infection models that other IFNα subtypes (e.g., IFNα14) are much more potent in restricting HBV replication than IFNα2 (3). IFNα14 does that by eliciting effective type I and II signaling crosstalk inducing many potent antiviral effector molecules (3). Especially in HIV infection, direct antiviral treatment regimens (combined antiretroviral therapy, cART) are quite efficient in keeping the virus in check; however, functional cure is still an obstacle in HIV therapeutic approaches. Thus, efficient activation of the host immune response, in particular cytotoxic effector responses of T cells and natural killer (NK) cells, are most likely required for a sustained control of HIV infection. We have previously shown that IFNα14, IFNα6, and IFNα17 significantly inhibited HIV replication in infected gut-derived lamina propria mononuclear cells (LPMCs), the earliest cellular targets during natural HIV infection, as well as peripheral blood mononuclear cells (PBMCs), whereas the clinically approved IFNα2 showed only moderate antiviral effects against HIV in both cell types (2, 10). Detailed analysis in HIV-infected humanized BLT (bone marrow/liver/thymus) mice *in vivo* further confirmed this superior antiviral effect of IFNα14, which significantly reduced viral loads and increased expression of HIV restriction factors (2). Interestingly, IFNα2 treatment increased activation of CD8^+^ T cells and expression of T cell exhaustion markers (2, 11), whereas IFNα14 targeted NK cell effector responses and did not increase the expression of exhaustion markers on CD8^+^ T cells in HIV-infected humanized mice. Gene therapy with plasmids encoding IFNβ and IFNα14, but not the clinically used IFNα2, confers long-term suppression of HIV-1 replication in HIV-infected humanized mice (12).

Nevertheless, a comprehensive understanding of the biological effects of IFNα2 in comparison to the other type I IFN subtypes on human immune cells during HIV infection is still missing. Here, we aim to close this knowledge gap by conducting a systematic investigation of the immunomodulatory properties of different type I IFNs on the important target cell populations LPMCs and PBMCs during HIV infection. Specifically, the gut is a critical site for early HIV-1 infection, as well as for driving chronic immune activation (13). Here, we infected LPMCs and PBMCs from healthy donors with HIV-1 *in vitro* and we further utilized PBMCs from HIV-infected individuals (People living with HIV; PLWH) and intensively studied the effect of different type I IFN subtypes on innate and adaptive immune cells in these samples. The analysis of these cell cultures highlighted significant differences in expression of cytotoxic molecules after stimulation with different type I IFNs. We showed that IFNα14 and IFNβ rather than IFNα2 induced the expansion of CD107a^+^ gut-derived CD4^+^ T cells as well as blood-derived T cells and reduced the proportions of IFNγ-expressing T cells. Additionally, downstream analyses reveal cell source and tissue-specific differences upon stimulation with different type I IFNs. In summary, we could demonstrate that IFNα14 and IFNβ mostly increased cytotoxic T cell responses and decreased IFNγ responses during HIV infection; nevertheless, chronic hyperimmune activation was not driven by type I IFNs.

## Material and Methods

### Study Participants/Isolation and Cultivation of Primary Cells

All HIV^+^ blood samples (6 male HIV^+^ individuals under cART; HIV-1 RNA copies < 40/ml; the average age was 53.7 years +/- 5.6 years) were collected from the Department of Dermatology at the University Hospital Essen; healthy HIV^-^ samples (n=6) were donated by healthy individuals of the University Hospital Essen. Blood collection was approved by the Ethics Committee (No.:11-4715) of the University of Duisburg-Essen.

PBMCs were isolated from each blood sample by density gradient centrifugation. For this purpose, 9 ml of EDTA-whole blood mixed with RPMI 1640 supplemented with 100 U/ml penicillin and 100 mg/ml streptomycin was layered on Pancoll solution (Pan Biotech, Aidenbach, Germany) and centrifuged at 900 x g for 35 minutes with brakes off. Then, the PBMCs (interphase) were transferred to a new 50 ml tube and washed twice with RPMI 1640 medium supplemented with penicillin/streptomycin. Cryostocks with 1x10^7^ PBMCs/ml were prepared in fetal calf serum (FCS) (Sigma Aldrich, St. Louis, USA) supplemented with 10% DMSO.

PBMCs were thawed one day prior to experiments. Up to 90% of viable cells were cultivated in RPMI 1640 with 10% FCS, 100 U/ml penicillin, 100 mg/ml streptomycin, 2 mM L-glutamine, and 10 mM HEPES. Cells were incubated at a density of 1x10^6^ cells/ml over night at 37°C, 5% CO_2_.

LPMCs were isolated from patients undergoing abdominal surgery (diagnoses: neuroendocrine tumor (terminal ileum), colon (ascending colon) cancer; refractory bleeding from the small intestine; oesophagogastric junctional adenocarcinoma; malignant melanoma with metastases in small intestine) at the University Hospital Essen. Samples were received *via* the Westdeutsche Biobank, pathologically evaluated, and judged macroscopically normal and healthy. LPMC collection was approved by the Ethics Committee (No.:15-6310) of the University of Duisburg-Essen. Gut samples from 6 different patients were used in this study, 3 females and 3 males. The average age was 64.3 years (+/-17 years). In order to isolate LPMCs, samples were processed as previously described (14). Briefly, mucosal tissue was rinsed with HBSS and treated with 1.6 mM DTT for 40 min at 37°C. The tissue was rinsed twice with HBSS, and the epithelium was removed with two 60 min treatments of 1 mM EDTA in HBSS and 0.1% BSA (AppliChem, Darmstadt, Germany) at 37°C. Afterwards, mucosal tissue was minced into 1-2 mm square portions and treated with 1-2 mg/ml of collagenase D (Sigma Aldrich) in RPMI 1640 for two additional 60 min treatments. LPMCs from each treatment were passed through a cell strainer, and stored in RPMI 1640 with 45% FCS and 10% DMSO in liquid nitrogen.

LPMCs were thawed one day prior to experiments. Up to 50% of viable cells were cultivated in RPMI 1640 with 10% human serum (Type AB, Pan Biotech), 100 U/ml penicillin and 100 mg/ml streptomycin, 1% L-glutamine and 0.4% piperacillin/tazobactam (Fresenius Kabi, Bad Homburg an der Hoehe, Germany). Cells were incubated at a density of 2.5x10^6^ cells/ml over night at 37° C, 5% CO_2_.

### Stimulation With Different Human IFNα Subtypes and IFNβ

IFNβ was commercially acquired from PBL assays sciences, whereas IFNα subtypes were produced and purified as previously described (15). Briefly, recombinant IFNs were expressed in *E. coli* after M13 phage transduction. To harvest the proteins, the bacteria were pelleted, the protein-containing inclusion bodies were denatured by sonication, dissolved in 6M guanidin-hydrochlorid, and refolded in arginine. The recombinant proteins were further purified by ion exchange chromatography and size exclusion chromatography, specificity and purity of the proteins were verified after each step *via* an SDS gel. By phase separation of the products with Triton X-114, remaining endotoxin was removed from the solution. Endotoxin levels were tested using ToxinSensor (GenScript, Piscataway, USA) and are below 0.25 EU/mL. The activity of each subtype was determined using the human ISRE-Luc reporter cell line, a retinal pigment epithelial cell line transfected with a plasmid containing the Firefly Luciferase gene, stably integrated under control of the IFN-stimulation-response element (ISRE). Following stimulation with type I IFNs, chemiluminescence can be detected and used to calculate the respective activity in units against commercially available IFNα subtypes (PBL assays sciences) (2).

### Infection

X4-HIV-1_NL4-3-IRES-Ren_ reporter virus was produced by transfection of HEK293T cells with pNL4.3Ren (16). The TCID_50_ were calculated by X-Gal staining of infected TZM-bl reporter cells.

LPMCs were mock-treated or infected with multiplicity of infection (MOI) of 0.25 *via* spinoculation at 1,200 x g for 2 h. Viral input was removed and cells were washed with DPBS. LPMCs were cultivated in fresh media containing 2000 U/ml of the appropriate IFNs at a density of 1x10^6^ cells/ml. Supernatant of HIV-infected and mock-treated cells were collected at the indicated time points and stored at -80°C until further use. Additionally, cells were lysed with PJK lysis buffer (PJK, Kleinblittersdorf, Germany) for the determination of viral loads.

PBMCs were thawed one day prior to infection and activated with 1 µg/ml PHA in presence of 10 ng/ml IL-2 (Miltenyi Biotec, Bergisch Gladbach, Germany) at a density of 1x10^6^ cells/ml over night at 37°C, 5% CO_2_. All other conditions for the infection were the same as for LPMC infection.

### Cell Surface and Intracellular Staining by Flow Cytometry

For surface staining, cells were washed once with FACS buffer (PBS containing 0.1% BSA and 0.02% sodium azide) and cells were incubated for 15 min with the antibody mixture in FACS buffer. Cell surface staining was performed using the following antibodies: anti-CD3 (UCHT1, eBioscience™), anti-CD4 (RPA-T4, BioLegend, San Diego, USA), anti-CD8 (RPA-T8, BioLegend), anti-CD56 (HCD56, BioLegend), anti-HLA-DR (L243, BioLegend), anti-CD107a (H4A3, BioLegend), and anti-CD253/Tumor Necrosis Factor Related Apoptosis Inducing Ligand (TRAIL) (RIK-2, BD). The Fixable Viability Dye (FVD) eFluor™ 780 (eBioscience™) was used to exclude dead cells from the analysis. Cells were washed with FACS buffer and fixated with 4% PFA for up to 2 h. Cells were washed twice with Intracellular Staining Perm Wash Buffer (BioLegend) and incubated for 20 min with anti-GzmB (GB11, BD) and anti-IFNγ (XNG1.2, BD) in Intracellular Staining Perm Wash Buffer. Cells were washed again twice with Intracellular Staining Perm Wash buffer, collected in FACS staining buffer, and stored at 4°C until acquisition. Samples were acquired with a BD LSR II flow cytometer with a HTS module and data were analyzed using FACSDiva and FlowJo Version 10.8 (both BD Becton Dickinson, Heidelberg, Germany).

### Phosphoflow Staining

For phosphoflow analysis cells were stimulated with 2000 U/ml IFNα2, IFNα14, IFNβ, or left unstimulated (-IFN) for 15 min at 37°C. Surface staining was performed simultaneously to IFN stimulation with the following antibodies: anti-CD3 (UCHT1, eBioscience™), anti-CD4 (RPA-T4, BioLegend), anti-CD8 (RPA-T8, BioLegend), anti-CD56 (HCD56, BioLegend), as well as FVD. After stimulation cells were immediately fixated with pre-warmed Fixation Buffer (BioLegend) at 37°C. Cells were then permeabilized with pre-chilled TruePhos™ Perm Buffer (BioLegend) at -20°C for 1 h. Subsequently, cells were washed twice with Intracellular Staining Perm Wash buffer and the following antibodies were added to the cells: anti-STAT1 pTyr702 (Miltenyi Biotec); anti-STAT3 pSer727 (Miltenyi Biotec), and anti-STAT3 pTyr705 (eBioscience™), anti-STAT5 pTyr694 (BioLegend). After a 30 min incubation, cells were washed twice with FACS Intracellular Staining Perm Wash buffer and stored at 4°C until acquisition. Samples were acquired with a BD LSR II flow cytometer with a HTS module and data were analyzed using FACSDiva and FlowJo Version 10.8.

### Stimulation With SEB and HLA Class I Restricted HIV Peptide Pool

LPMCs and PBMCs from healthy individuals and PBMCs from cART-treated PLWH were stimulated either with 200 ng/ml SEB (Merck, Darmstadt, Germany) or with 1 µg/ml of an HLA class I restricted HIV peptide pool (consisting of 22 peptides, each corresponding to a defined HLA class I-restricted T cell epitope from HIV; PE peptides & elephants GmbH, Potsdam, Germany) in the presence of 2000 U/ml IFNα2, IFNα14, IFNβ, or without IFN (-IFN) for 4 days. PBMCs were re-stimulated with 5 µg/ml SEB or 1 µg/ml peptide pool and incubated in presence of antibodies against the co-stimulatory molecules CD28 (9F10, BioLegend) and CD49d (CD2.2, BioLegend) at 37°C for 6 h. Brefeldin A with a final concentration of 5 µg/ml was added after 1 h of stimulation. Supernatants were collected and stored at -80°C until further use and cells were immediately used for flow cytometric analysis.

### Multiplex Cytokine and Chemokine Bead Arrays

Cytokine and chemokine quantification of supernatants from HIV-infected und uninfected cell cultures were done using the LEGENDplex COVID-19 Cytokine Storm multiplex assay 1 (BioLegend) according to the provider’s instructions and analyzed using FACSDiva and the dedicated software provided by BioLegend. Data acquisition was performed on a BD LSRII flow cytometer with HTS module.

### Statistical Analysis

Experimental data were reported as means ± SEM. Statistically significant differences between the all groups were analyzed using Friedman test and Dunn’s multiple comparison test. Statistical analyses were performed using GraphPad Prism software v8 (GraphPad, San Diego, CA, USA).

## Results

### Stimulation of *In Vitro* HIV-Infected Primary Cell Cultures With Different Type I IFN Subtypes Elevated Cytotoxic T Cell Responses

**Figure 1:**
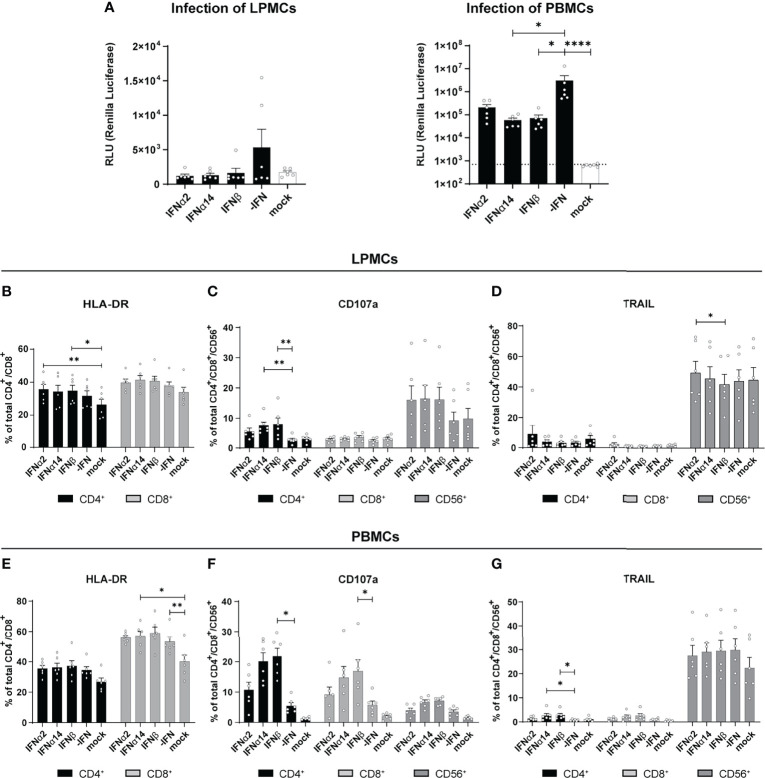
Immune cell activation and cytokine expression after IFN treatment of *in vitro* infected LPMCs and PBMCs with HIV. LPMCs and PBMCs from healthy individuals were exposed to X4-HIV-1_NL4-3-IRES-Ren_ or mock treated with medium and subsequently treated with 2000 U/ml IFNα2, IFNα14, or IFNβ. Cells were lysed 4 dpi and viral loads were determined by the relative light units (RLUs). **(A)** Inhibition of HIV replication by different IFNs *in vitro.* Additionally, flow cytometric analysis was performed to analyze immune responses by IFNα2, IFNα14, and IFNβ. **(B–D)** Frequencies of HLA-DR^+^, CD107a^+^, and TRAIL^+^ T and/or NK cells in LPMCs. **(E–G)** Frequencies of HLA-DR^+^, CD107a^+^, and TRAIL^+^ T and/or NK cells in PBMCs. Mean values ± SEM are shown for n=6. Statistical analyses between the treated groups within a cell population were done by using Friedman test and Dunn’s multiple comparison test. ****, P < 0.0001; **, P < 0.01; *, P < 0.05.

Type I IFNs are critical effector cytokines during most viral infections and their pleiotropic biological functions are important to fully control viral infections. In particular during chronic HIV infection, the induction of hyperimmune activation of T cells by increased IFN signatures is controversially discussed (17–23). In chronically HIV-infected humanized mice the type I IFN signature was associated with T cell dysfunction and reduced viral control (24–26). In order to further scrutinize the immunomodulatory effects of type I IFNs during HIV infection we utilized different primary cells such as blood-derived PBMCs and gut-derived LPMCs as a model of HIV target immune cell interaction during natural infection. We infected these cells cultures *in vitro* with a Renilla Luciferase expressing X4-tropic HIV_NL4.3._ In addition, the infected cell cultures were directly stimulated with IFNα2, IFNα14, or IFNβ and the IFN-mediated effects on viral loads, immune cell activation, as well as cytokine and chemokine production were analyzed four days post infection (dpi). We chose these three IFNs as IFNα14 was previously shown to have the highest antiviral activity against HIV-1 (2, 10–12), IFNα2 is the clinically approved IFNα subtype with only moderate anti-HIV activity, and IFNβ, as it has the highest affinity to both subunits of the IFNα/β receptor (IFNAR1/2) resulting in vigorous activation of downstream signaling events (6, 27). In addition, IFNβ might be involved in chronic immune activation during persistent HIV infection (7) and chronic Lymphocytic choriomeningitis virus (LCMV) infection (28). As shown in **Figure 1A** treatment with the different IFNs resulted in decreased infection of LPMCs shown by Renilla Luciferase activity in cell lysates, which is in line with previous reports (2, 12). In HIV-infected PBMCs treatment with IFNα14 and IFNβ significantly reduced viral titers, whereas the effect of IFNα2 was less pronounced (**Figure 1A**). In HIV-infected humanized mice we previously saw an increased activation and effector response of CD8^+^ T cells upon IFNα2 treatment and an improved NK cell cytotoxicity after treatment with IFNα14. Thus, we next elucidated the effect of the different IFNs on T cell activation and NK/T cell cytotoxic effector functions in our HIV-infected primary cell cultures. In *in vitro* infected LPMCs slightly increased frequencies of HLA-DR-expressing T cells were observed, but no additional effect of IFNs was detected (**Figure 1B**). Interestingly, the proportion of CD4^+^ T cells expressing the degranulation marker CD107a, as a surrogate marker for cytotoxicity, was significantly higher after IFN treatment compared to untreated cells (-IFN; **Figure 1C**). Furthermore, the frequencies of CD107a^+^ CD4^+^ T cells increased most after IFNα14 and IFNβ stimulation. In HIV-infected PBMCs the effects of IFN treatment on CD107a^+^ T cells was even stronger compared to HIV-infected LPMCs (**Figure 1F**). In contrast to studies from HIV-infected humanized mice (2, 11) *in vitro* stimulation with IFNα14 and IFNβ significantly increased percentages of CD107a-expressing CD4^+^ and CD8^+^ T cells in comparison to untreated and IFNα2-treated HIV-infected PBMCs. Similar effects were also observed in CD56^+^ NK cells, whereas the changes in CD107a-expressing CD56^+^ NK cells were not significant in LPMCs and PBMCs (**Figures 1C, F**). Only minor immunomodulatory effects of IFN stimulation on TRAIL-expressing cells in LPMCs (**Figure 1D**) as well as HLA-DR- and TRAIL-expressing cells in PBMCs (**Figures 1E, G**) were observed.

**Figure 2:**
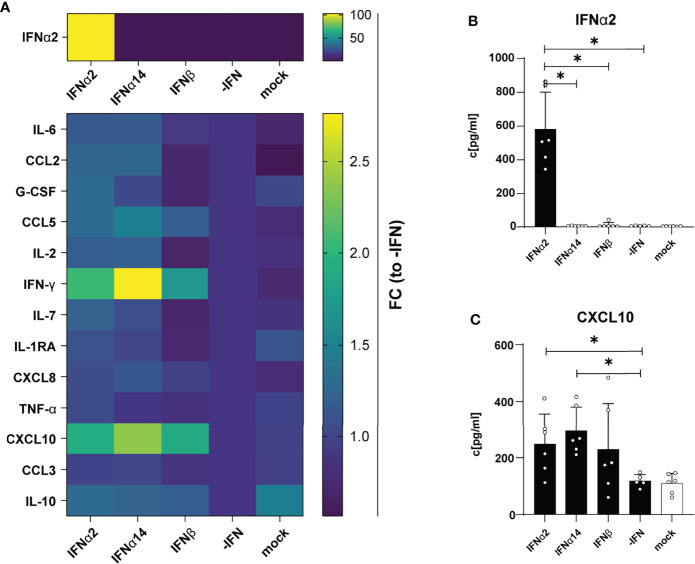
Cytokine and chemokine profile in supernatants from HIV- and mock-infected LPMCs after different IFN treatment. LPMCs were infected with X4-HIV-1_NL4-3-IRES-Ren_ or mock treated with medium and treated with 2000 U/ml IFNα2, IFNα14, IFNβ, or without IFN (-IFN). Supernatants were harvested 4 dpi and 14 cytokines and chemokines were analyzed simultaneously by a multiplex bead-based assay. **(A)** Averaged fold changes (FC) of each individual donor of IFNα2, IL-6, CCL2, G-CSF, CCL5, IL-2, IFNγ, IL-7, IL-1RA, CXCL8, TNFα, CXCL10, CCL3, and IL-10 expression of HIV-infected LPMCs after stimulation with type I IFN subtypes relative to HIV-infected, non-treated LPMCs (-IFN). **(B, C)** Absolute quantification of IFNα2, and CXCL10 in supernatants of HIV-infected LPMCs treated with IFNα2, IFNα14, IFNβ, or without IFN (-IFN). Mean values ± SEM are shown for n=6. Statistical analyses between the treated groups within a cell population were performed by using Friedman test and Dunn’s multiple comparison test. *, P < 0.05.

Next, we were interested in the chemokine and cytokine production in HIV-infected LPMC cultures and how they are affected by different IFNs. Thus, we analyzed 14 different chemokines and cytokines in the supernatants of IFN-stimulated and HIV-infected LPMCs at 4 dpi. The cytokines IL-2, IL-7, IL-10, and TNFα were not detected above baseline and no difference after IFN-stimulation were found for CCL3, IL-6, IL-1RA, G-CSF, CCL2 and CXCL8. As depicted in **Figures 2A, B**, HIV infection itself did not induce any IFNα2 and also the treatment with IFNα14 and IFNβ did not result in the expression of IFNα2 by an IFN-mediated feedback loop suggesting that we just detected the IFNα2 that was added for stimulation. Furthermore, the stimulation with IFNs led to increased expression of the IFN-induced protein CXCL10 (**Figures 2A, C**, **S1**). In conclusion, IFNα14 and IFNβ increased the percentages of cytotoxic CD4^+^ T cells in infected LPMCs as well as CD107a-expressing CD4^+^ and CD8^+^ T cells in HIV-infected PBMCs, whereas the overall effect of IFNα2 was only moderate. Further, IFNα14 and IFNβ increased the expression of distinct cytokines and chemokines in HIV-infected LPMCs.

### Additional Type I IFN Stimulation of LPMCs and PBMCs had Only Minor Effects on T and NK Cell Responses

**Figure 3:**
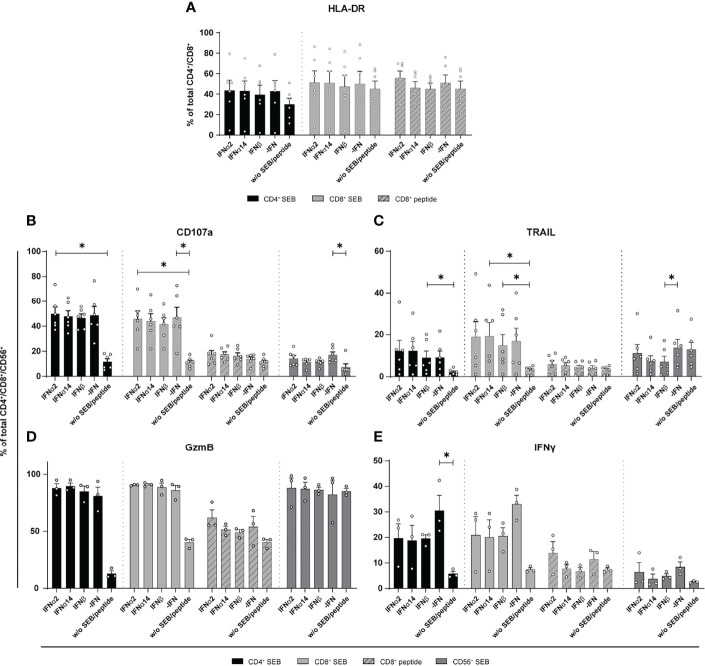
Expression of cytotoxic molecules by *in vitro* stimulated T and NK cells of PBMCs from cART treated PLWH. PBMCs from cART-treated PLWH were stimulated either with 200 ng/ml SEB or with 1 µg/ml of an HLA class I restricted HIV peptide pool in presence of 2000 U/ml IFNα2, IFNα14, IFNβ, or without IFN (-IFN) for 4 days. PBMCs were re-stimulated with 5 µg/ml SEB or 1 µg/ml peptide pool respectively and incubated in presence of antibodies against the co-stimulatory molecules CD28 and CD49d for 6 h BFA was added after 1 h of stimulation. Flow cytometry was used to analyze T cell activation and cytokine expression. **(A)** Activation profile determined by the frequencies of HLA-DR^+^ CD4^+^ and CD8^+^ T cells with or without stimulation in the presence or absence of the different IFNs. **(B–E)** Frequencies of the cytotoxic molecules CD107a, TRAIL, GzmB, and IFNγ expressed on CD4^+^, CD8^+^ T cells, and CD56^+^ NK cells. Mean values ± SEM are shown for **(A–C)** n=6 and **(D, E)** n=3. Statistical analyses between the treated groups within a cell population were done by using Friedman test and Dunn’s multiple comparison test. *, P < 0.05.

Stimulation with different type I IFNs resulted in distinct immunomodulatory effects in cultures of *in vitro* HIV-infected LPMCs and PBMCs. Thus, we wanted to elucidate if NK and T cells from chronically HIV-infected individuals can be activated by type I IFN stimulation. Thus, PBMCs from cART-treated PLWH were stimulated with either staphylococcal enterotoxin B (SEB) in order to trigger polyclonal T cell and NK cell activation (29) or an HLA class I-restricted HIV peptide pool for 4 days in the presence and absence of the different IFNs. At day 4 post stimulation the cells were re-stimulated with SEB or the HIV peptide pool, and analyzed by flow cytometry. As shown in **Figure 3** stimulation with the different type I IFNs did not change frequencies or expression levels (MFI; data not shown) of activated T cells (**Figure 3A**). Treatment with the different IFNs did not change the percentages of CD107a, TRAIL, and GzmB-expressing cells in comparison to untreated cells (**Figures 3B–D**). Percentages of TRAIL-expressing CD56^+^ NK cells were significantly reduced upon IFNβ-stimulation (**Figure 3C**). IFN stimulation tended to reduce the frequency in IFNγ-producing cells from PLWH, but this effect was not significant (**Figure 3E**).

**Figure 4:**
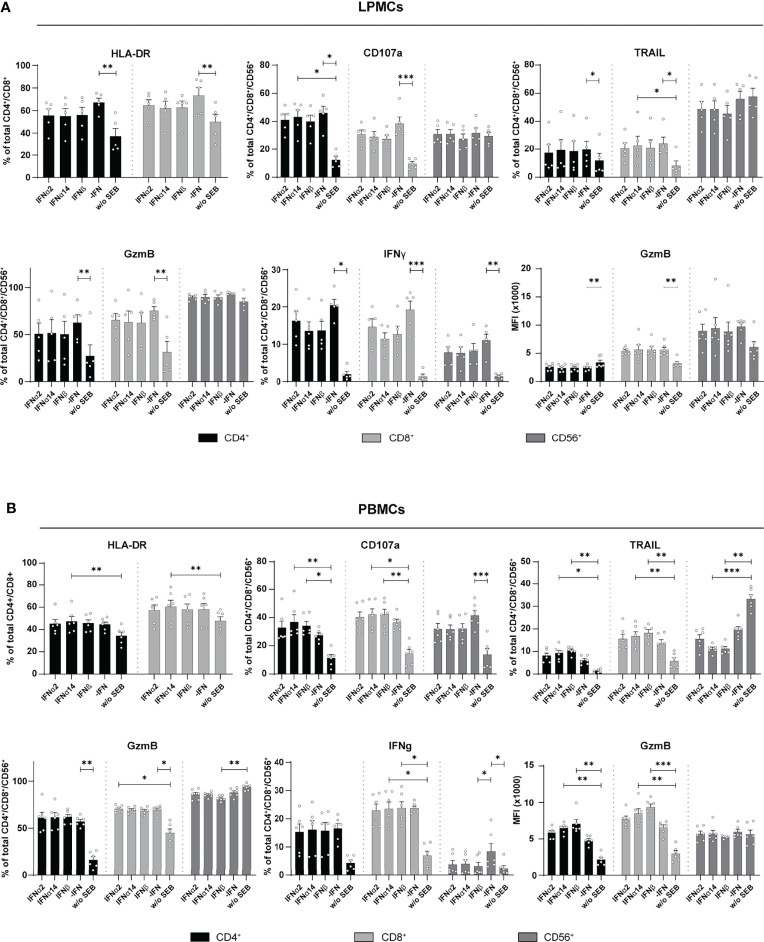
Cytokine expression after IFN treatment of LPMCs, and PBMCs from healthy individuals. **(A)** LPMCs and **(B)** PBMCs from healthy individuals were stimulated as described before with SEB and treated with either 2000 U/ml IFNα2, IFNα14, IFNβ, or without IFN (-IFN). frequencies of HLA-DR, CD107a, TRAIL, GzmB, and IFNγ expressing cells as well as GzmB expression per cell shown as MFI after treatment of SEB-treated PBMCs and LPMCs. Mean values ± SEM are shown for **(A)** n=5 and **(B)** n=6. Statistical analyses were done by using Friedman test and Dunn’s multiple comparison test. ***, P < 0.001; **, P < 0.01; *, P < 0.05.

The overall immunomodulatory effect of IFN stimulation on PBMCs from HIV-infected individuals was quite low. We next wanted to decipher if these minor IFN-mediated effects on PBMCs from chronically HIV-infected individuals were affected by the virus itself. Therefore, LPMCs derived from the gut of healthy individuals, which is a critical site for early HIV-1 infection and a driver of chronic immune activation, were similarly stimulated by type I IFNs for 4 days with SEB and the activation and effector functions of T and CD56^+^ NK cells in the culture were analyzed. Similar to PBMCs from PLWH, further stimulation with type I IFN subtypes did not change the percentages of activated and cytotoxic T and CD56^+^ NK cells in LPMCs from healthy individuals (**Figure 4A**). Again, frequencies of IFNγ-expressing T cells were reduced in IFN-stimulated T cells and the reduction was even stronger after stimulation with IFNα14 or IFNβ; however, the observed effect was not significant (**Figure 4A**). Next, we analyzed PBMCs from healthy individuals after stimulation with SEB in the presence and absence of the different type I IFNs. In line with the previous results from LPMCs from healthy individuals and PBMCs from chronically HIV-infected individuals, IFN stimulation of primary healthy PBMCs did not change percentages of activated T cells. However, frequencies of cytotoxic CD4^+^ T cells (CD107a^+^, TRAIL^+^) and their expression level of GzmB (MFI) were significantly increased upon stimulation with IFNβ and IFNα14 (**Figure 4B**). Interestingly, percentages of TRAIL-expressing CD56^+^ NK cells were significantly reduced after stimulation with IFNα14 and IFNβ and no changes were found on IFNγ-expressing T cells after IFN stimulation. Taken together, we could show that an additional stimulation with different type I IFN subtypes had only minor effects on T and NK cell responses from primary gut-derived LPMCs and PLWH PBMCs. In contrast, healthy PBMCs responded stronger to IFNα14 and IFNβ by elevated cytotoxic T cell responses.

**Figure 5:**
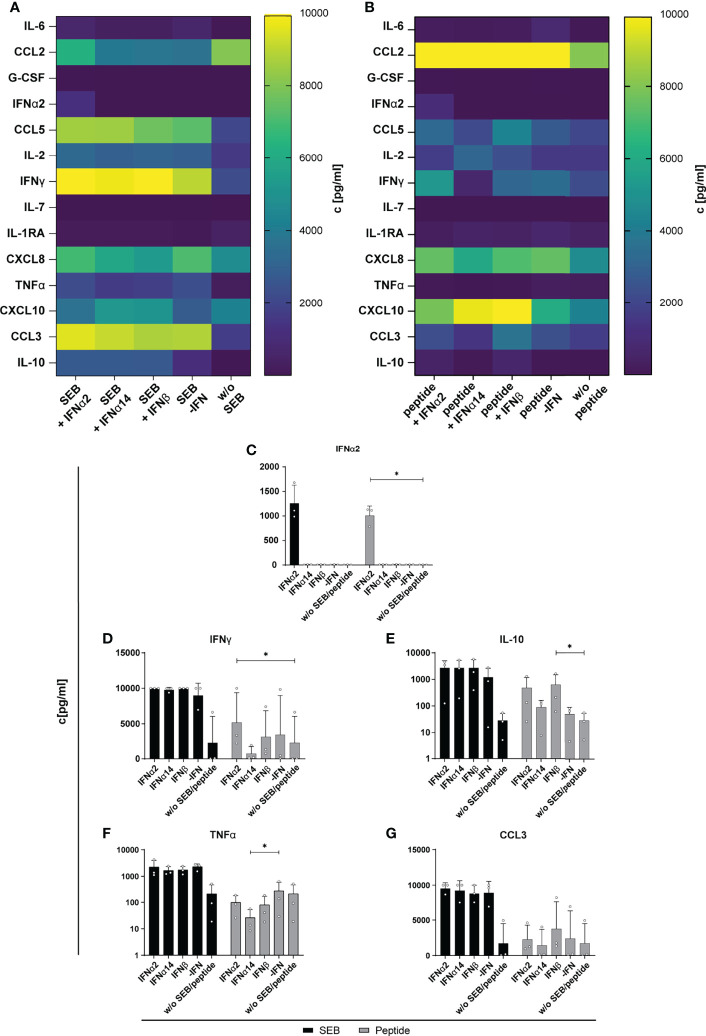
Cytokine and chemokine profile in supernatants of SEB and HIV peptide pool stimulated PBMCs from PLWH. PBMCs from cART-treated PLWH were stimulated as described in **Figure 3** with SEB or HIV peptide pool and treated with either 2000 U/ml IFNα2, IFNα14, IFNβ, or without IFN (-IFN). On day 4 supernatants were collected for multiplex bead-based assay to quantify cytokines and chemokines. **(A)** Heatmap of IL-6, CCL2, G-CSF, IFNα2, CCL5, IL-2, IFNγ, IL-7, IL-1RA, CXCL8, TNFα, CXCL10, CCL3, and IL-10 concentrations in pg/ml after stimulation with SEB or **(B)** HIV peptide pool. **(C–G)** Concentration of IFNα2, IFNγ, IL-10, TNFα, and CCL3 in supernatants of SEB or HIV peptide pool stimulated PBMCs from PLWH after different IFN treatment. Mean values ± SEM are shown for n=3. Statistical analyses between the treated groups within a cell population were done by Friedman test and Dunn’s multiple comparison test. *, P < 0.05.

Next, we determined the effect of IFN stimulation on cytokines and chemokines expression in the supernatant of SEB- and HIV-peptide pool stimulated PBMCs from PLWH. Thus, we measured 14 different cytokines and chemokines as mentioned before. All tested cytokines and chemokines were above baseline levels. As depicted in **Figure 5**, SEB and peptide stimulation themselves did not result in IFNα2 induction and again treatment with IFNα14 and IFNβ did not result in any IFNα2 production suggesting that again we detected only the IFNα2 that was added for stimulation. Both stimulations (SEB and HIV peptide pool) strongly induced the expression of CCL2, CCL5, IFNγ, CXCL8 (IL-8), and CCL3, which was not affected by treatment with the different IFNs. Only IL-10 expression in the supernatant slightly increased upon stimulation with the different type I IFNs. However, this effect was not significant, and we did not observe any difference between the IFN subtypes (**Figure 5E**). Taken together, IFN stimulation of PBMCs from HIV-infected individuals did not affect the expression of the analyzed immune effector molecules including chemokines, IFNs, interleukins, colony-stimulating factors, and TNFα.

### IFNα14 and IFNβ Strongly Induced Phosphorylation of STAT1 and STAT5 in Immune Cell Subsets

**Figure 6:**
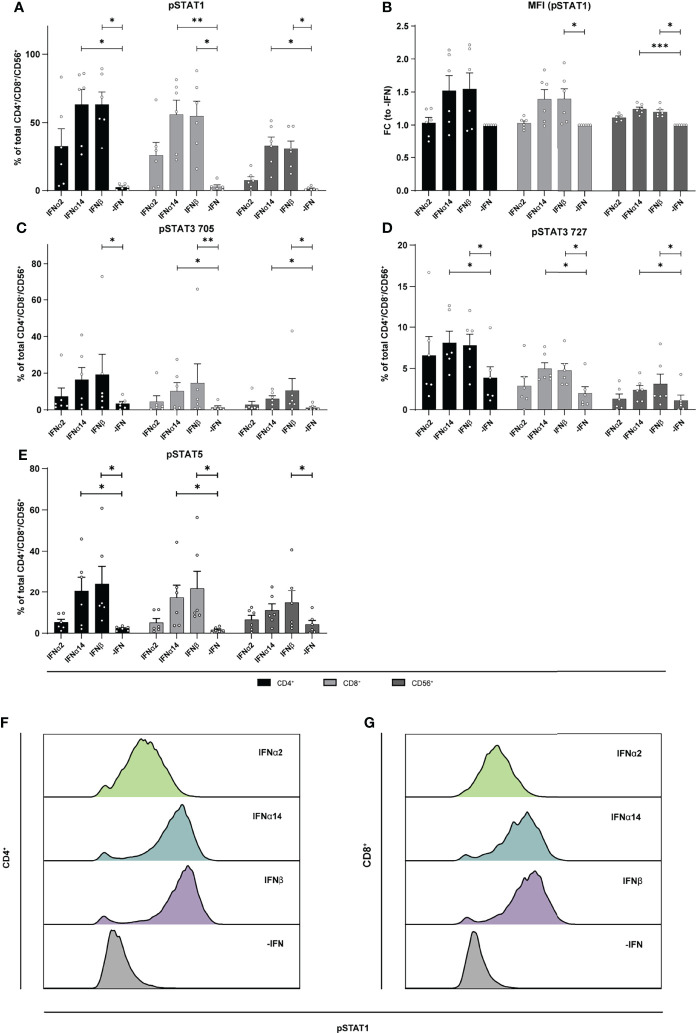
Phosphorylation of STAT molecules after IFN stimulation in LPMCs. LPMCs were stimulated with 2000 U/ml IFNα2, IFNα14, IFNβ, or without IFN (-IFN) in presence of the surface markers anti-CD3, anti-CD4, anti-CD8, anti-CD56, and the viability marker FVD. Cells were then fixated and permeabilized for phosphoflow analysis with anti-STAT1 pTyr702, anti-STAT5 pTyr694, anti-STAT3 pTyr705, and anti-STAT3 pSer727. **(A)** Frequencies of phosphorylated STAT1, **(B)** mean donor-specific fold changes of MFI (pSTAT1) **(C)** frequencies of phosphorylated STAT3 pTyr705, **(D)** STAT3 pSer727, and **(E)** STAT5 on CD4^+^, CD8^+^ T, and NK cells. Mean values ± SEM are shown for n=6. Statistical analyses between the treated groups within a cell population were done by using Friedman test and Dunn’s multiple comparison test. ***, P < 0.001; **, P < 0.01; *, P < 0.05. **(F)** Representative histograms of phosphorylated STAT1 expression by unstimulated or IFN-stimulated CD4^+^ and **(G)** CD8^+^ T cells.

To elucidate the underlying molecular mechanism of the different effector responses after IFN treatment of the two cell populations (LPMCs and PBMCs), we performed phosphoflow analysis with LPMCs and PBMCs from healthy and HIV-infected patients upon stimulation with the different IFNs. Thus, we stimulated PBMCs from PLWH, and PBMCs and LPMCs from healthy individuals with the different IFNs for 15 min and analyzed the phosphorylation of the immune cell signaling molecules STAT1, 3, and 5 on T and CD56^+^ NK cells. In uninfected LPMCs, stimulation with the different IFNs increased the frequency of T and CD56^+^ NK cells expressing the different phosphorylated STAT molecules with IFNα14 and IFNβ inducing higher phosphorylation compared to IFNα2. However, for IFNα14 and IFNβ the strongest effect on phosphorylation of STAT1^+^ in T and CD56^+^ NK cells was observed (**Figure 6**). Interestingly, frequencies of pSTAT1^+^ CD4^+^ and CD8^+^ T cells were higher than frequencies of pSTAT1^+^ CD56^+^ NK cells.

**Figure 7:**
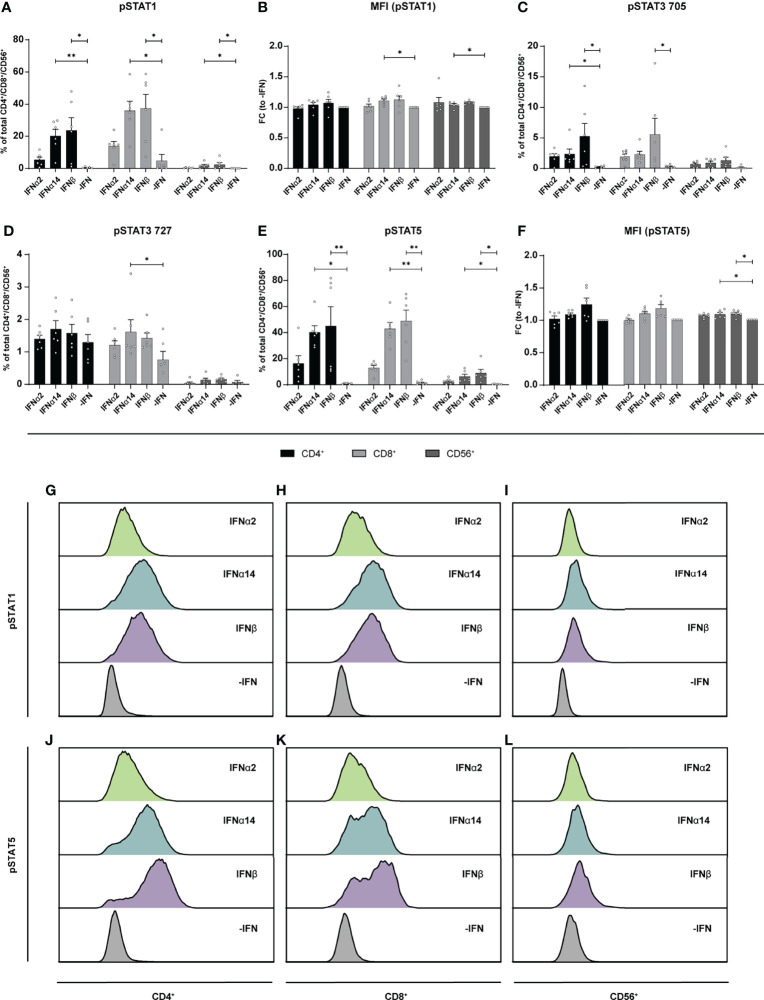
Phosphorylation of STAT molecules after IFN stimulation in PBMCs. PBMCs from healthy donors were stimulated with 2000 U/ml IFNα2, IFNα14, IFNβ, or without IFN (-IFN) in presence of the surface markers anti-CD3, anti-CD4, anti-CD8, anti-CD56, and the viability marker FVD. Cells were then fixated and permeabilized for phosphostaining with anti-STAT1 pTyr702, anti-STAT5 pTyr694, anti-STAT3 pTyr705, and anti-STAT3 pSer727. **(A)** Frequencies of phosphorylated STAT1, **(B)** mean donor-specific fold changes of MFI (pSTAT1) **(C)** frequencies of phosphorylated STAT3 pTyr705, **(D)** STAT3 pSer727, **(E)** STAT5, and **(F)** mean donor-specific fold changes of MFI (pSTAT5) on CD4^+^, CD8^+^ T, and NK cells. Mean values ± SEM are shown for n=6. Statistical analyses between the treated groups within a cell population were done by using Friedman test and Dunn’s multiple comparison test. **, P < 0.01; *, P < 0.05. **(G)** Representative histograms of phosphorylated STAT1 expression by unstimulated or IFN-stimulated CD4^+^, **(H)** CD8^+^ T cells, and **(I)** NK cells as well as **(J)** phosphorylated STAT5 expression by CD4^+^, **(K)** CD8^+^ T cells, and (L) NK cells.

**Figure 8:**
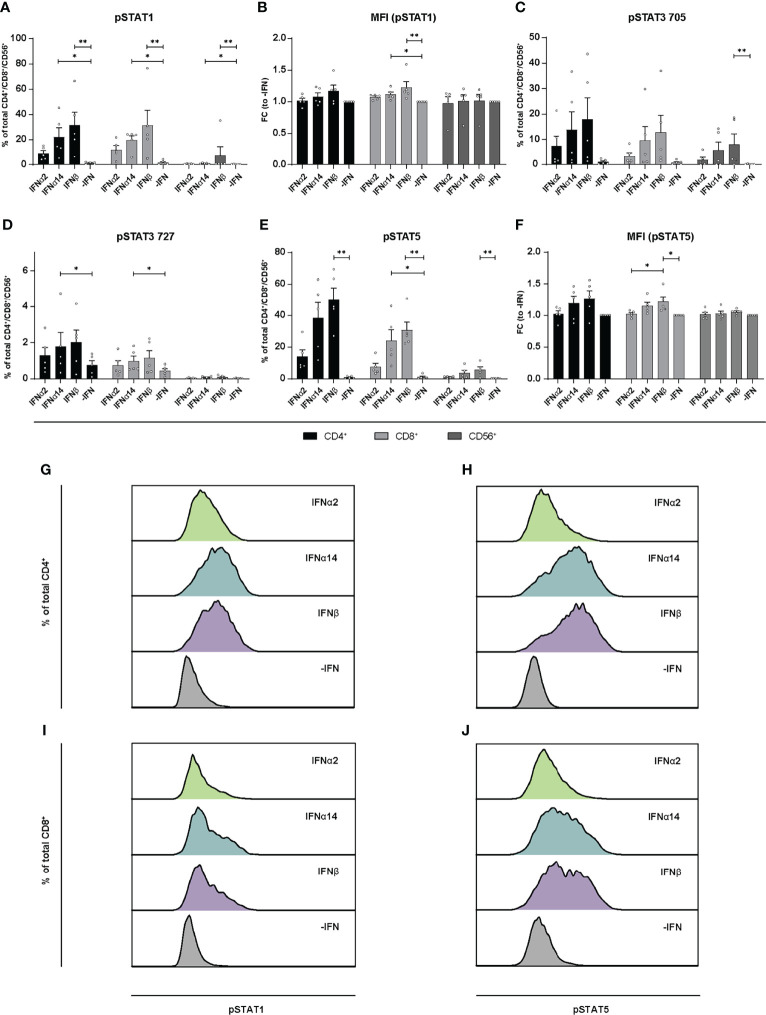
Phosphorylation of STAT molecules after IFN stimulation in PBMCs from PLWH. PBMCs from PLWH were stimulated with 2000 U/ml IFNα2, IFNα14, IFNβ, or without IFN (-IFN) in presence of the surface markers anti-CD3, anti-CD4, anti-CD8, anti-CD56, and the viability marker FVD. Cells were then fixated and permeabilized for phosphostaining with anti-STAT1 pTyr702, anti-STAT5 pTyr694, anti-STAT3 pTyr705, and anti-STAT3 pSer727. **(A)** Frequencies of phosphorylated STAT1, **(B)** mean donor-specific fold changes of MFI (pSTAT1) **(C)** frequencies of phosphorylated STAT3 pTyr705, **(D)** STAT3 pSer727, **(E)** STAT5, and **(F)** mean donor-specific fold changes of MFI (pSTAT5) on CD4^+^, CD8^+^ T, and NK cells. Mean values ± SEM are shown for n=5. Statistical analyses between the treated groups within a cell population were done by using Friedman test and Dunn’s multiple comparison test. **, P < 0.01; *, P < 0.05. **(G, H)** Representative histograms of phosphorylated STAT1 and STAT5 expression by unstimulated or IFN-stimulated by CD4^+^ T cells, and **(I, J)** CD8^+^ T cells.

Similar to the LPMCs described above (**Figure 6**), phosphoflow analysis of IFN-stimulated PBMCs from healthy individuals (**Figure 7**) and PLWH (**Figure 8**) also showed increased frequencies of pSTAT1^+^ T cells after IFNα14 and IFNβ stimulation, but not after IFNα2 stimulation (**Figures 7A, B**, **8A, B**). In addition, significantly higher proportions of pSTAT3^+^ and pSTAT5^+^ T cells were found and were again highest after IFNα14 and IFNβ treatment (**Figures 7C–F**, **8C–F**). Overall, frequencies of pSTAT1/3/5^+^ CD56^+^ NK cells were remarkably low in contrast to T cells (**Figures 7**, **8**) as well as to CD56^+^ NK cells in LPMCs (**Figure 6**). The phosphoflow analysis confirmed differences between type I IFN subtypes in activating downstream signaling cascades, which might cause the observed differences in T cell and NK cell effector functions.

Taken together, we could show that stimulation with IFNα2, IFNα14, and IFNβ modulated T cell responses during HIV-infection, which might be regulated by early signaling events. Overall, IFNβ and IFNα14 stimulation resulted in similar immunomodulatory responses, whereas the stimulatory effect of IFNα2 was weaker. Additionally, the source of immune cells (PBMCs from blood, LPMCs from gut) is associated with differences in activation of immune cells and their effector functions upon IFN stimulation during HIV infection.

## Discussion

Type I IFNs are well-known cytokines eliciting broad antiviral effects by inducing the expression of antiviral genes called ISGs, some of which can directly inhibit HIV replication. It has been shown that the initial induction of type I IFNs is extremely important in controlling the acute phase of HIV-1 infection and to limit reservoir size and disease course (2, 12). However, elevated IFN signatures during persistent HIV infection can be detrimental and might contribute to enhanced systemic inflammation (30). Here, we dissected the complex effects that IFNs have on T and NK cells during HIV infection, with investigations of their downstream signaling events. Our data revealed that stimulation with three different type I IFN subtypes inhibited HIV replication *in vitro* [**Figures 1A** and (2, 10)]; however, their immunomodulatory properties were indeed quite diverse. IFNα14 and IFNβ significantly increased percentages of CD107a-expressing CD4^+^ T cells from *in vitro* HIV-infected LPMCs and T and NK cells from *in vitro* HIV-infected PBMCs. In comparison with these two subtypes IFNα2 showed significantly reduced frequencies of CD107a-expressing immune cells (**Figures 1C, F**), which was also observed for CXCL10 expression in these cultures (**Figures 2A–C**). In contrast, IFNα2 treatment increased the proportions of activated virus-specific CD8^+^ T cells which was differentially affected by IFNα14 and IFNβ in PBMCs from PLWH (**Figure 3A**). Interestingly, IFNγ responses were suppressed in polyclonal and HIV peptide pool stimulated T and NK cells by all type I IFNs. Comprehensive phosphoflow analysis unveiled strong differences in phosphorylation of STAT1 and STAT5 by the different IFN subtypes with IFNβ and IFNα14 having the highest activating potency. Interestingly, the phosphorylation pattern of IFN-stimulated immune cells from healthy LPMC cultures showed distinct signaling events in comparison to PBMCs from healthy and HIV-infected individuals.

To uncover the exact role of type I IFNs in persistent HIV infection, their broad biological responses have to be investigated. The right choice of the perfect experimental model is important to elucidate the IFN-response in humans. As mice cannot be infected with HIV-1 due to the lack of entry receptors and co-receptors, many studies were either done in HIV-infected humanized mice or in SIV (Simian immunodeficiency virus)-infected rhesus macaques. The experimental data are still puzzling as the different studies showed quite opposing results. We and others have previously shown that during acute and chronic HIV infection of BLT-humanized mice, IFNα14 treatment resulted in significantly reduced viremia and the treatment did not induce hyperimmune activation (2, 11, 12, 31). During acute HIV infection treatment with IFNα2 resulted in increased frequencies of CD8^+^ T cells expressing cytotoxic molecules like CD107a and Granzyme B, and IFNα14 preferentially targeted NK cell responses by increasing TRAIL expression (2). Further, IFNα14 treatment during chronic HIV infection in humanized mice was associated with reduced expression of markers for T cell dysfunction, whereas IFNα2 treatment did not changed the expression rate of exhaustion markers, indicating that exogenous treatment with IFNα2 may not efficiently prevent T cell exhaustion in HIV infection (11). IFN-blocking experiments in persistently HIV-1 infected humanized mice demonstrated that despite having increased viral loads upon blockade, IFNAR signaling may still drive CD4^+^ T cell apoptosis and dysfunction of CD4^+^ and CD8^+^ T cells during chronic infection (25). Additionally, others reported that cART therapy combined with IFNAR blockade in HIV-1 infected humanized mice decreased plasma RNA levels as well as numbers of latently infected cells (24). This study demonstrated that persistent type I IFN signaling during the chronic phase of HIV infection may help to dampen viral replication although it also contributes to the depletion of CD4^+^ T cells. It has to be taken into account that studies with exogenous human IFNs or human IFNAR blocking experiments have limitations in humanized mice. Not all aspects of the complex human immune system are entirely recapitulated in these mice. Apart from the human immune cells, different mouse cells like epithelial or endothelial cells are still present in humanized mice, which differentially respond to treatments targeting human cells, making it difficult to completely transfer the knowledge from those infection studies or immunotherapies into the human system. SIV infection in rhesus macaques is another well-established model to elucidate the beneficial or detrimental role of type I IFNs in acute and chronic retrovirus infection. During acute and chronic SIV infection, type I IFNs are important for viral control and to limit viral reservoir as shown by a human type I IFN antagonist (32, 33). Application of human IFNα2a prior to SIV infection of rhesus macaques initially inhibited systemic infection due to strong ISG upregulation; however, prolonged administration resulted in type I IFN desensitization, reduced ISG expression, increased SIV reservoir size, and lower CD4^+^ T cell counts compared to untreated controls. This suggests that the timing and kinetics of IFN treatments have to be carefully analyzed in HIV or SIV infection *in vivo*. Another important aspect which has to be considered is that the IFN-antagonist and the human IFNα2a, that was used for the studies is human and not rhesus macaque-specific. That might also change the binding and the effector response as it is known that the amino acid sequences of the IFNs and their receptor differ between different species. Moreover, it is known from the human IFNα system that small differences in the amino acid sequence also change the binding affinity and the ISG expression pattern, as described for the human IFNα subtypes (3, 27). Thus, upcoming studies in monkeys should consider the species-specific IFNs, antagonists or blocking antibodies to minimize unwanted responses.

In our study we described IFN subtype-specific differences in the modulation of T and NK-cell responses during HIV infection. In most experiments, we observed quite similar results for IFNα14 and IFNβ, both known for their high binding affinity to their IFN receptors. IFNα2b binds IFNAR1 and IFNAR2 with lower binding affinities (6) which is more than 5-fold and 2-fold weaker compared to IFNα14. IFNβ was shown to have the tightest affinity to both receptor subunits (27), which was thought to correlate with effector responses (6, 27). The binding affinity is an important factor influencing the IFN-effector responses, but, also other factors modulate the response like the avidity, timing of the response, cytokine milieu, or cell type are important [reviewed in (34)]. In chronic LCMV infection IFNα was shown to control early viral dissemination, but it does not affect persistent viral infection (28). Interestingly, blocking of IFNβ but not IFNα improved antiviral T cell responses and reduced viral loads during persistent LCMV infection by decreasing the amounts of infected DCs. Similar differences between IFNα and IFNβ were also observed in Chikungunya virus infection (35) and Friend retrovirus infection (36). Interestingly, only IFNα2a/b is approved for clinical treatment against chronic viral infections, whereas IFNβ is only used therapeutically against multiple sclerosis (MS), but not as an antiviral drug.

Another aspect of hyperimmune activation and T cell exhaustion during HIV infection are elevated expression levels of CXCL10, IL-10 and PD-1. All of these factors have been shown to be IFN-inducible, thus the effects of IFN stimulation or treatments *in vitro* and *vivo* have to address their expression profile. CXCL10 was strongly induced during HIV infection (37, 38), and stimulation with IFNα14 decreased CXCL10 levels in HIV-infected humanized mice (2). It has been reported that plasma CXCL10 levels are associated with rapid disease progression in early HIV-1 infection (39) and increased levels in plasma and small intestine are associated with a more rapid HIV/SIV disease onset after infection (40). In addition, elevated plasma CXCL10 levels positively correlated with HIV viral load and viral reservoir size (41). Furthermore, T cell functions in PLWH on cART were inhibited by CXCL10 (42). In our study we observed a slight increase in CXCL10 expression by *in vitro* HIV-infected LPMCs (**Figures 2A, C**) and polyclonal and peptide-stimulated immune cells from PLWH (**Figure 5**) after stimulation with the different IFNs thus increased CXCL10 levels are associated with type I IFN subtype stimulation. The expression of negative regulators like IL-10 is also described during chronic HIV infections and blockade of the IL-10 pathway enhances HIV-1-specific CD4^+^ T cell functions (43–45). In our previous study with humanized mouse, we did not detect any effect of type I IFNs on IL-10 levels, which is in line with the data of the current study (**Figures 2**, **5**), suggesting that IL-10 is not regulated by type 1 IFN. In addition, the observed effects of different type I IFN subtypes on CXCL10 expression during HIV infection were quite low and the infection kinetics were different for *in vitro* infections compared to natural infection. Thus, the influence of type I IFNs on chemokines like CXCL10 during HIV infection has to be addressed in more detail.

During HBV infection *in vitro* it was already shown that IFNα14 in contrast to IFNα2 elicited type I and type II IFN signaling shown by the activation of the IFNγ-activated sequence as well as STAT1 homodimerization. IFNα14 also exclusively phosphorylated STAT6 (3), demonstrating its pleiotropic biological response during HBV infection. Here, we found that apart from the classical Jak-STAT signaling (phosphorylation of STAT1) also other non-classical pathways like STAT3 or STAT5 (46–49) were activated by phosphorylation upon stimulation with the different type I IFNs. We observed that IFNβ and IFNα14 induced the strongest STAT1 and STAT5 phosphorylation, which was much weaker in IFNα2-treated cells (**Figures 6**–**8**). Interestingly, cell type-specific differences were observed with gut-derived LPMCs showing higher activation of the classical downstream signaling cascades, whereas PBMCs either from healthy or HIV-infected patients showed a higher activation of the non-classical signaling pathway STAT5. In the context of HIV infection, STAT5 was shown to bind to the HIV LTR promotor increasing HIV transcription (50) and HIV production is enhanced in primary CD4^+^ T cells following STAT5 activation (51). HIV-1 is also able to stimulate STAT3 activation in primary human macrophages *via* Nef (52), and HIV-1 Tat expression increases STAT3 expression and phosphorylation in astrocytes (53) providing evidence for STAT3 as an effector in HIV-infected cells. STAT3 was also recognized as critical regulator of dendritic cell function and physiology, making this factor quite important for the analysis of IFN-modulated T and NK cell responses which are initiated by dendritic cells (5). In particular during HIV-infection binding of gp120 to CCR5 promotes STAT3 activation in dendritic cells leading to IL-6 induction (54). We also found an IFN-mediated phosphorylation of STAT3 at Tyr705, but this effect was not significant compared to non-IFN treated samples (**Figures 6**–**8**). Interestingly, PBMCs from PLWH had initially higher frequencies of pSTAT3 Tyr705 expressing immune cells in comparisons to healthy controls suggesting an increased STAT3 activation during HIV infection. Further studies on how the different type I IFN subtypes modulate immune responses during acute and chronic HIV-1 infection, how the different IFN-mediated downstream signaling events are regulated during infection and if chronic hyperimmune activation is triggered by different type I IFNs remain to be investigated to develop new treatment approaches against chronic HIV infection.

## Data Availability Statement

The original contributions presented in the study are included in the article/**Supplementary Material**. Further inquiries can be directed to the corresponding authors.

## Ethics Statement

The studies involving human participants were reviewed and approved by Ethics Committee (No.: 11-4715, No. 15-6310) of the University Hospital Essen. The patients/participants provided their written informed consent to participate in this study.

## Author Contributions

KS and UD conceived the study. ZK and MS performed experiments, statistical analysis and data analysis. CK and SE contributed to the design and implementation of the research. KS and ZK wrote the original manuscript. All authors edited and approved the final manuscript.

## Funding

This work was supported by the DFG SPP1923 to KS (SU1030/1-2) and UD (DI714/18-2).

## Conflict of Interest

The authors declare that the research was conducted in the absence of any commercial or financial relationships that could be construed as a potential conflict of interest.

## Publisher’s Note

All claims expressed in this article are solely those of the authors and do not necessarily represent those of their affiliated organizations, or those of the publisher, the editors and the reviewers. Any product that may be evaluated in this article, or claim that may be made by its manufacturer, is not guaranteed or endorsed by the publisher.

## Supplementary Material

Supplementary Figure 1 Cytokine and chemokine concentrations in supernatants from HIV- and mock-infected LPMCs after treatment with distinct type I IFN subtypes LPMCs were infected with X4-HIV-1_NL4-3-IRES-Ren_ and treated with 2000 U/ml IFNα2, IFNα14, IFNβ, or remained untreated (-IFN). Supernatants were harvested 4 dpi and IFNα2, CCL5, IL-2, IFNγ, IL-7, IL-1RA, TNFα, CXCL10, CCL3, and IL-10 (top) as well as IL-6, CCL2,G-CSF, and CXCL8 (bottom) were analyzed simultaneously by a multiplex bead-based assay. Concentrations of the different detected Cytokines and chemokines are depicted in a heatmap. Mean values ± SEM are shown for n=6.
