# Supplementary figures and images for: Distinct Type I Interferon Subtypes Differentially Stimulate T Cell Responses in HIV-1-Infected Individuals
Source: Front Immunol. 2022 Jul 13;13:936918. doi: 10.3389/fimmu.2022.936918 (PMC9326074; doi:10.3389/fimmu.2022.936918)

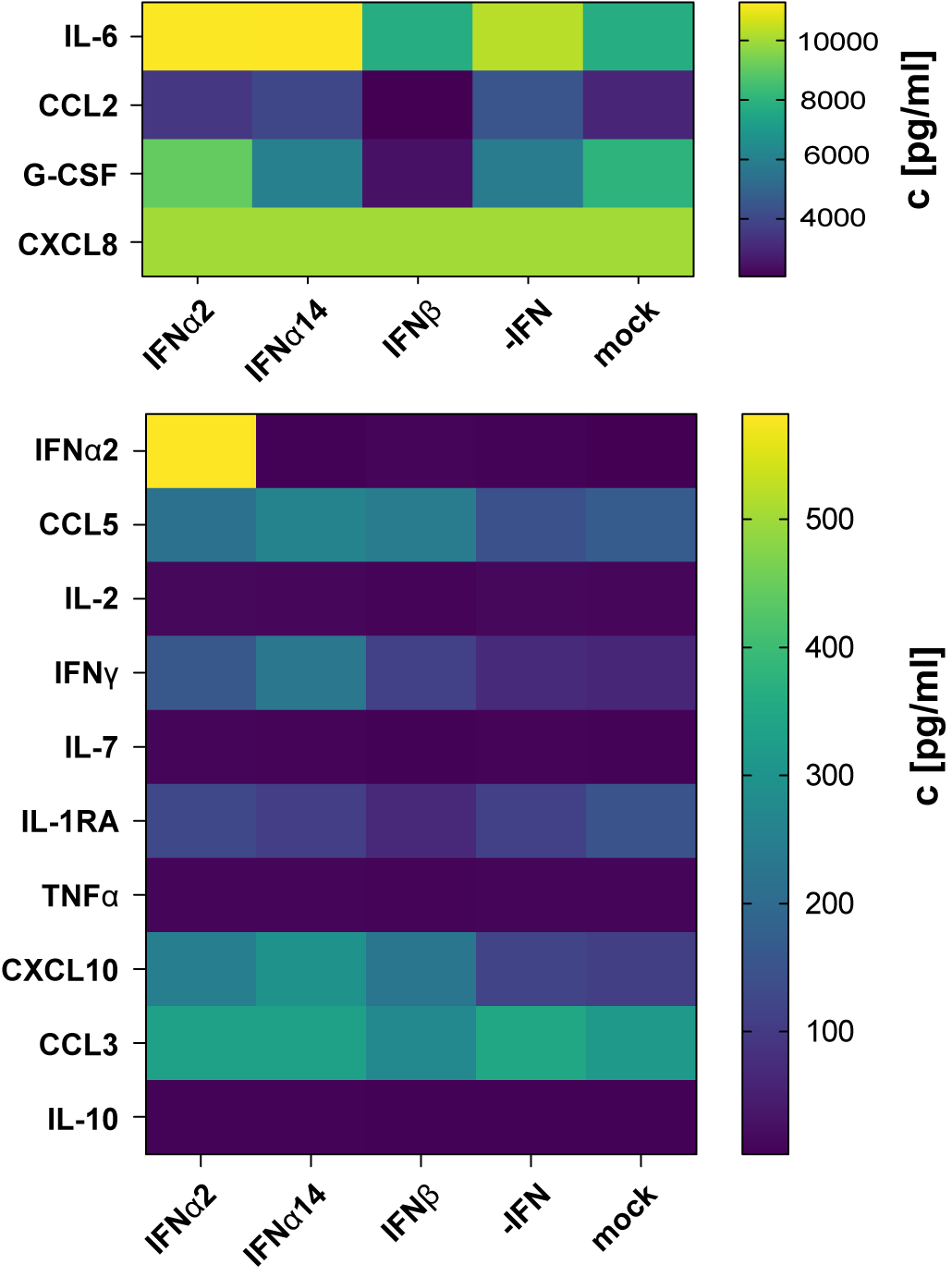

Supplement: Supplementary Figure 1 — Cytokine and chemokine concentrations in supernatants from HIV- and mock-infected LPMCs after treatment with distinct type I IFN subtypes LPMCs were infected with X4-HIV-1NL4-3-IRES-Ren and treated with 2000 U/ml IFNα2, IFNα14, IFNβ, or remained untreated (-IFN). Supernatants were harvested 4 dpi and IFNα2, CCL5, IL-2, IFNγ, IL-7, IL-1RA, TNFα, CXCL10, CCL3, and IL-10 (top) as well as IL-6, CCL2,G-CSF, and CXCL8 (bottom) were analyzed simultaneously by a multiplex bead-based assay. Concentrations of the different detected Cytokines and chemokines are depicted in a heatmap. Mean values ± SEM are shown for n=6. [file Image_1.tif]
